# Respond to the letter to the editor by Van’t Hul et al. regarding the published manuscript “can do, don’t do” are not the lazy ones: a longitudinal study on physical functioning in patients with COPD” by Sievi et al.(1)

**DOI:** 10.1186/s12931-020-01376-2

**Published:** 2020-05-13

**Authors:** Noriane A. Sievi, Malcolm Kohler, Christian F. Clarenbach

**Affiliations:** University Hospital Zurich, Zurich, Switzerland

We want to thank the authors for the letter and their interest in our study [1]. They raise several important points.

The relationship between physical activity (PA) and physical capacity (PC) in patients with chronic obstructive pulmonary disease (COPD) is controversially discussed, although the negative impact of physical inactivity in these patients is a stable finding in the literature [2, 3]. Therefore, a concept that includes PA and PC in relation to each other, such as the one developed by Koolen et al. [4], could provide a very useful and practicable tool to identify clinical characteristics responsible for the impairments observed in PA and/or PC. Such a concept may also allow improving the effect of physical functioning interventions. We applied this concept in our COPD cohort to challenge its robustness and stability. We feel that drawing definitive conclusions regarding its usability will require more cohorts than just two. Furthermore, the additional application of the PC-PA concept on longitudinal data was possibly not the intention of the inventors. Due to the longitudinal data we could also assess if patients mainly remain in a PC-PA quadrant over several years or if there are annual quadrant changes. Changes in PC and/or PA are frequently observed in COPD patients. Therefore, the concept was also tested for its ability to reflect these changes. We are aware of the fact that changes in PC-PA quadrants can be due to various changes of physical functioning, as Van’t Hul et al. mention. We are convinced that due to the usual natural history of COPD, all patients will eventually approach the “can’t do, don’t do” situation and the quadrants are located on this downward slope starting with “can do, do do”. Based on the results from our previous study showing a decline in PA while PC remained stable, we conclude that decrease in PC occurs time delayed [5]. That is why we summarized all subjects with either decreasing PA or PC or both in one group. Since there are no studies that observed COPD patients long enough to proof this hypothesis, there is the possibility that our approach may be inappropriate.

Van’t Hul et al. correctly state that we used a different equation for the percentage of prediction of the 6-min walking distance (6MWD) and that methodology is always a key factor for comparability of different studies and we should have considered this. However, if the use of another, also widely used equation, generates completely different results in quadrant phenotypes, it remains questionable, if the concept, in its present form, is able to describe the underlying mechanisms categorizing the patients into the four PC-PA quadrants. The same is true for differences in primary versus secondary care or disease severity, which was indeed slightly different in our cohort.

We agree that Koolen et al. [4] never used the term “lazy” to characterize a subgroup of COPD patients. Instead, they describe the “can do, don’t do” quadrant as persons who have the ability to be active, but “just don’t do it” and state that promoting PA in this subgroup is not an “easy task”. To disentangle behavioral and physical aspects of inactivity will be the challenge for further research.
